# Supplementary material for: Genomic Characterization of Potential Plant Growth-Promoting Features of Sphingomonas Strains Isolated from the International Space Station
Source: Microbiol Spectr. 2022 Jan 12;10(1):e01994-21. doi: 10.1128/spectrum.01994-21 (PMC8754149; doi:10.1128/spectrum.01994-21)
Supplement: SUPPLEMENTAL FILE 2 — Supplemental material. Download SPECTRUM01994-21_Supp_1_seq11.pdf, PDF file, 0.3 MB [file spectrum01994-21_supp_1_seq11.pdf]

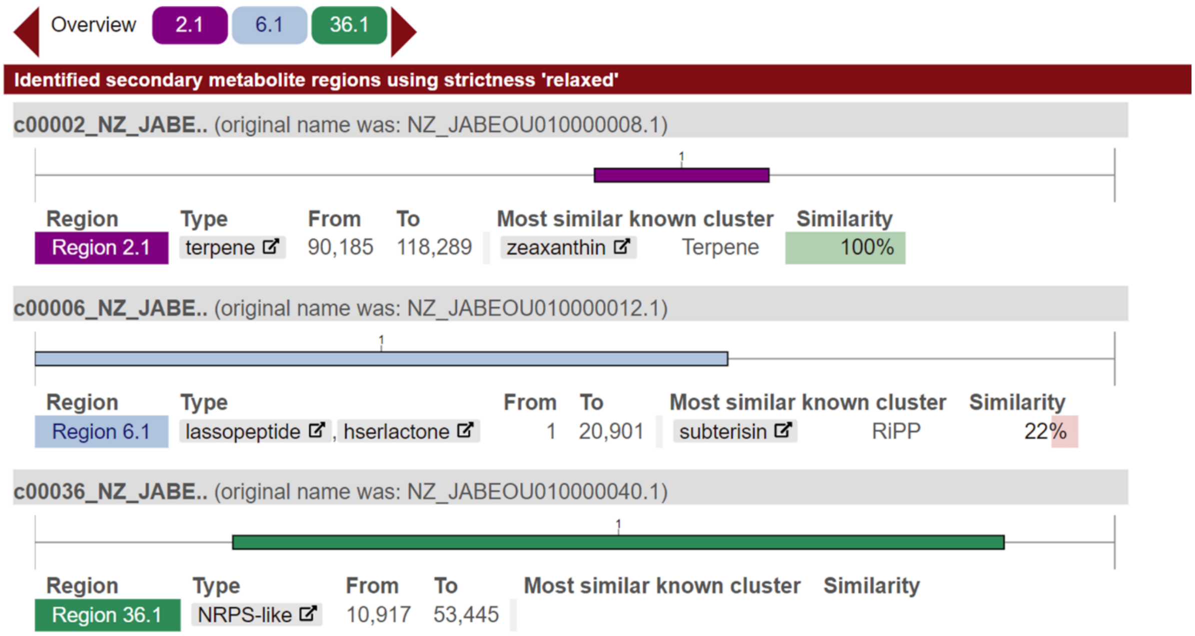

Supplemental Figure S1: Overview of biosynthetic clusters identified via antiSMASH-Bacteria online webserver (v5.2.0, strictness: relaxed, all features included), displaying a homoserine lactone (autoinducer) biosynthetic cluster (Region 6.1) that is likely unique to the spaceflight *S. paucimobilis* FKI-L5-BR-P1 genome.
